# Nanophytosomal Gel of *Heydotis corymbosa* (L.) Extract against Psoriasis: Characterisation, In Vitro and In Vivo Biological Activity

**DOI:** 10.3390/ph17020213

**Published:** 2024-02-06

**Authors:** Neelam Singh, Ayaz Mukarram Shaikh, Puneet Gupta, Béla Kovács, Mohammed F. Abuzinadah, Aftab Ahmad, Radha Goel, Swapnil Singh, Chaitanya Vinayak

**Affiliations:** 1 ITS College of Pharmacy, Ghaziabad 201206, Uttar Pradesh, India; singhneelam16@gmail.com (N.S.);; 2 Faculty of Agricultural and Food Sciences and Environmental Management, Institute of Food Science, University of Debrecen, 4032 Debrecen, Hungary; ayaz.shaikh@agr.unideb.hu (A.M.S.); kovacsb@agr.unideb.hu (B.K.); 3 Centre for Pharmaceutical Chemistry and Analysis, Amity Institute of Pharmacy, Amity University Uttar Pradesh, Sector 125, Noida 201313, UP, India; 4 Department of Medical Laboratory Sciences, Faculty of Applied Medical Sciences, King Abdulaziz University, Jeddah 21589, Saudi Arabia; mabuzinadah@kau.edu.sa; 5 Health Information Technology Department, The Applied College, King Abdulaziz University, Jeddah 21589, Saudi Arabia; 6 Pharmacovigilance and Medication Safety Unit, Centre of Research Excellence for Drug Research and Pharmaceutical Industries, King Abdulaziz University, Jeddah 21589, Saudi Arabia; 7 Lloyd Institutes of Management and Technology, Plot No.-11, Knowledge Park-II, Greater Noida 201306, UP, India; radhamit2006@gmail.com

**Keywords:** *Heydotis corymbosa*, nanogel, dermatokinetic, psoriasis, inflammatory markers, skin retention, plant derived compounds, in vitro and in vivo

## Abstract

The current study was conducted to examine the possible advantages of *Heydotis corymbosa* (L.) Lam. extract nanogel as a perspective for enhanced permeation and extended skin deposition in psoriasis-like dermatitis. Optimised nanophytosomes (NPs) were embedded in a pluronic gel base to obtain nanogel and tested ex vivo (skin penetration and dermatokinetics) and in vivo. The optimised NPs had a spherical form and entrapment efficiency of 73.05 ± 1.45% with a nanosized and zeta potential of 86.11 nm and −10.40 mV, respectively. Structural evaluations confirmed encapsulation of the drug in the NPs. Topical administration of prepared nanogel to a rat model of psoriasis-like dermatitis revealed its specific in vivo anti-psoriatic efficacy in terms of drug activity compared to the control and other formulations. Nanogel had improved skin integrity and downregulation of inflammatory cytokines. These findings suggest that developed phytoconstituent-based nanogel has the potential to alleviate psoriasis-like dermatitis with better skin retention and effectiveness.

## 1. Introduction

Inflammatory illnesses that are immune-mediated are a frequent and expanding issue. Patients who have diseases in one organ system frequently have co-morbidities in other organs, like the joint, eye, or skin, indicating that common processes are being activated across other organs [1]. Psoriasis is a non-contagious, autoimmune, inflammatory, and proliferative skin condition characterised by sharply distinct, peach-pink or drab-red thick patches with silvery scales that form specific skin lesions [2]. Together with discomfort and swelling, other disease-related symptoms include itchiness, skin peeling, and soreness. It is regarded as a chronic condition that affects individuals psychologically, physically, socially, and financially [3]. It also has unpredictable remissions and relapses [4], which affect any area of the body, although it is most commonly found on the scalp, lower back, and extensor surfaces of the limbs. However, it can manifest at any age, but most commonly between the ages of 15 and 22 and appears to reach a second peak between the ages of 60 and 69 [5]. Women are somewhat more likely than males to contract psoriasis at an earlier age, and family history also has a significant impact on when psoriasis first appears [5]. Psoriasis is a chronic, systemic inflammatory disease linked to the metabolic syndrome. Those with metabolic syndrome also have the cytokines that are braided in the pathophysiology of psoriasis [1,5]. Contrary to normal skin, psoriasis keratinocytes grow and spread rapidly from the basal layer to the skin’s surface within 6 to 8 days. Normally, keratinocytes develop and shed off every 35 to 40 days, but in the case of psoriasis, they mature and travel to the epidermis within a week, where they accumulate on the epidermis rather than being shed off [6]. Epidermal hyperproliferation, aberrant keratinocyte differentiation, acanthosis, angiogenesis with dilated dermal capillaries, and inflammatory cell infiltration in the skin layers are the histopathologic characteristics of psoriasis [7]. Although the precise cause of the illness is unclear, genetic, immunological, and predisposing factors may be involved. According to the severity of the condition, there are several lines of treatments that can be used topically, phototherapeutically, systemically, or biologically. However, none of them is ideal or safe [6,8]. For instance, hepatotoxicity and nephrotoxicity are linked to systemic use of methotrexate or cyclosporine, respectively [9,10]. When steroids are used topically, cardinal symptoms including skin shrinkage and hypopigmentation are observed. Some topical treatments might cause episodes of burning and itching [4]. The use of phototherapy has also been associated with certain negative consequences [11]. Despite the new biologics’ purported therapeutic effectiveness, which is made up of proteins and monoclonal antibodies, they are nonetheless more expensive than other treatments According to research [5] on the pathophysiology of psoriasis, a network of immune cells and their cytokines trigger a persistent inflammatory process [12]. Cytokine families include the interleukin (IL) family, the interferon (IFN) family, and the tumour necrosis factor (TNF) superfamily [12]. IL-1β, IL-2, IL-6,IL-10, IL-12, IL-22,IL-23, Th17/IL-17, TNF-α, IFN-γ cytokines are the major effector in the pathogenesis of psoriatic disease [13,14,15]. Monocyte chemotactic protein 1 (MCP-1/CCL2) has also recently been proposed as a possible biomarker to track the development of psoriasis [16,17]. Drugs that target these inflammatory markers are effective for treating the most prevalent kind of psoriasis, plaque psoriasis or psoriasis vulgaris (about 8 in 10 patients) [1,5].

The utilisation of phytoconstituents and their conversion to pharmaceutical dosage forms are crucial in the treatment of skin diseases since synthetic medications can lead to resistance and safety concerns [18]. Plants and secondary metabolites are vital in the development of novel, effective anti-psoriatic formulations. The mainstay of treating psoriasis is topical therapy and patients tolerate them well [19]. Target-based topical treatments are being developed in order to increase patient compliance, which is vital for the best results. In addition, progress in nanotechnology has opened the door to the prospect of enhancing topical agents’ efficacy by targeting and enhancing dosage forms’ adherence and/or drug penetration rates, along with reducing adverse effects [18]. Skin affected by psoriasis thickens abnormally, making effective topical administration of anti-psoriatic medications even more challenging. The capacity of nanoformulations to penetrate deeply into the dermis and epidermis of the skin results in increased accumulation in the skin layers and reduces systemic absorption and eventually, substantial improvement in psoriasis [20]. Compared to a raw herbal extract, its nanoformulation showed a high flux and retention of the drug in the skin layers [21]. Topical nanogels aid in drug delivery by hydrating and retaining the drug in the skin [5].

*Hedyotis corymbosa* (L.) Lam. Syn. *Oldenlandia corymbosa* (L.) Lam. (Rubiaceae) is a weedy herb, widely distributed throughout India. It is popularly referred to as “Parppatakapullu” in Keralan traditional medicine [22,23]. *Hedyotis corymbosa* (HC) is often used to treat bleeding, cancer, viral infections, acne, boils, and other skin conditions as well as appendicitis, hepatitis, and eye problems [22,23]. Previously, neuroprotective and anitinflammatory [24], antinociceptive [25], photokilling in skin cancer cells [23] and hepatoprotective activity [22,26] of HC on laboratory animals have also been studied [27].

The primary goal of this work was to prepare and optimise nanophytosomes using statistical design, and the optimised nanophytosomes were evaluated in vitro and ex vivo. Finally, they were fused with pluronic gel for topical administration, and a pre-clinical evaluation was carried out on an Imiquad (IMQ)-induced psoriasis rat model.

## 2. Results

### 2.1. Characterisation of Extract

Total flavonoid contents in the hydroalcoholic extract were (47.08 ± 0.44) mg quercetin equivalents/g dry weight. Total polyphenol content was 69.63 ± 3.56 mg Gallic acid/g. Polysaccharide content was estimated as 5.52 ± 0.78 mg pectin/g extract.

### 2.2. Evaluation of Prepared HC-NPs

#### 2.2.1. DSC Thermogram

**Figure 1:**
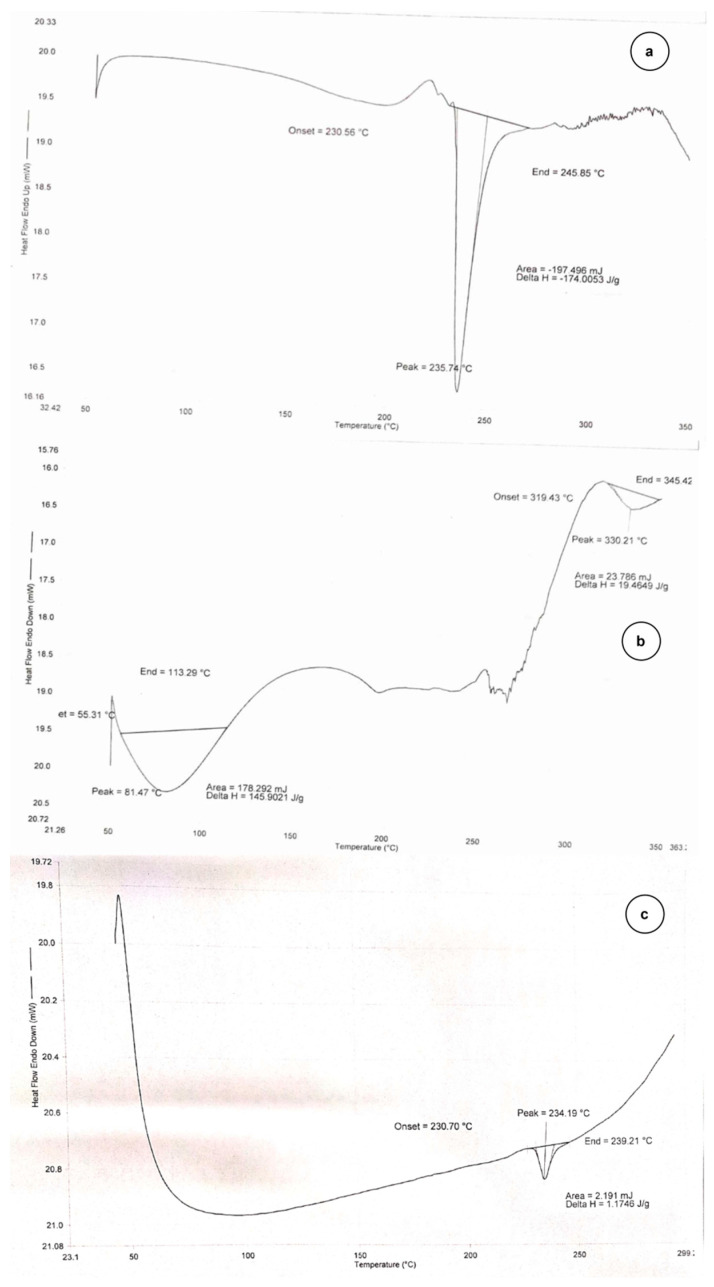
Representing Differential scanning colorimetry Thermogram of (**a**) Quercetin. (**b**) HC extract. (**c**) HC-NPs.

The thermal behavior of the pure quercetin and *Hedyotis corymbosa* extract nanophytosome (HC-NPs) are shown in Figure 1. All samples were first dehydrated by heating up to 120 °C. The DSC of pure quercetin showed an endothermic peak at 216 °C. This peak corresponds to melting as samples were already dehydrated in the first step. When guest moieties become entrapped in nanostructure pores, their boiling, melting, or sublimating points frequently shift or fade in DSC thermograms [28]. The endothermic peak of quercetin in HC-NPs faded, which indicates its encapsulation in the carrier lipid.

#### 2.2.2. Particle Size and Surface Charge

**Figure 2:**
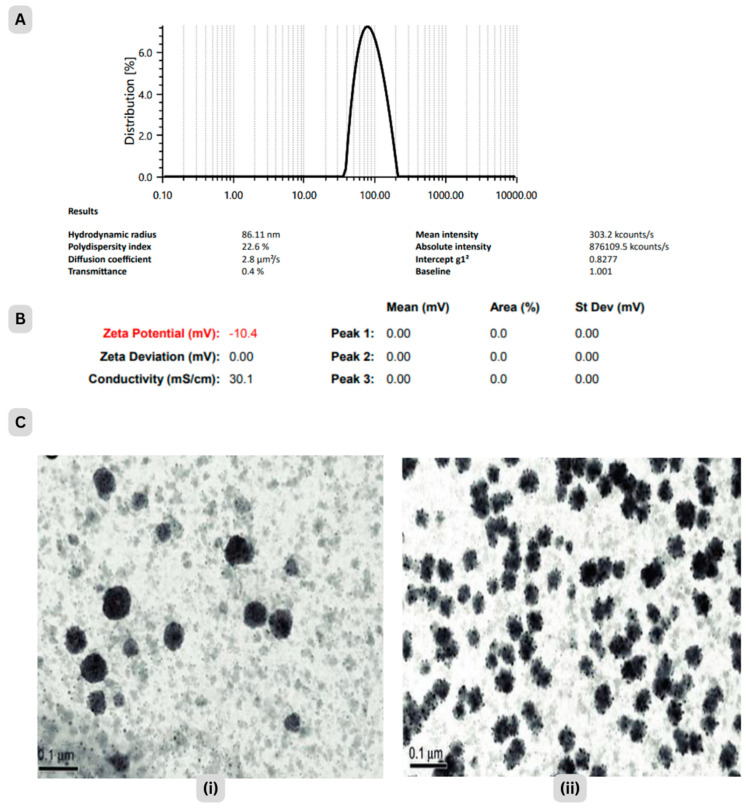
Representing (**A**) Particle size (**B**) surface charge and (**C**) TEM micrograph of optimized HC-NPs at (**i**) 30 min and (**ii**) 60 min ablation time.

The particle size of nanoparticles was observed as 86.11 ± 9.35 nm. Zeta potential at pH 6.5 was −10.40 mV, which could be attributed to increased repulsive forces and a lower tendency to aggregate, Figure 2A,B. PDI is basically a representation of the distribution of size populations within a given sample. Values of 0.2 and below are most commonly deemed acceptable in practice for nanoparticle materials. The homogeneous and stable form of nano-formulation was supported [29] by 0.116 PDI [28,29].

#### 2.2.3. Morphological Characterisation

Figure 2C shows Transmission Electron Microscope (TEM) images of HC-NPs at 30 min and 60 min ablation times. The photomicrograph of HC-NPs revealed that the nanophytosomes were spherical in shape and had a sealed structure [30].

#### 2.2.4. Entrapment Efficiency of HC-NPs

Entrapment efficiency provides an idea about the percentage of drugs that are effectively entrapped or adsorbed into nanophytosomes. Entrapment efficiency of HC-NPs was found in the range 73.05 ± 0.28%.

#### 2.2.5. Drug Content

The results obtained showed 89.78 ± 1.23% drug content in HC-NPs, indicating that the drug did not deteriorate during the formulation process [31].

#### 2.2.6. In Vitro Drug Release

**Figure 3:**
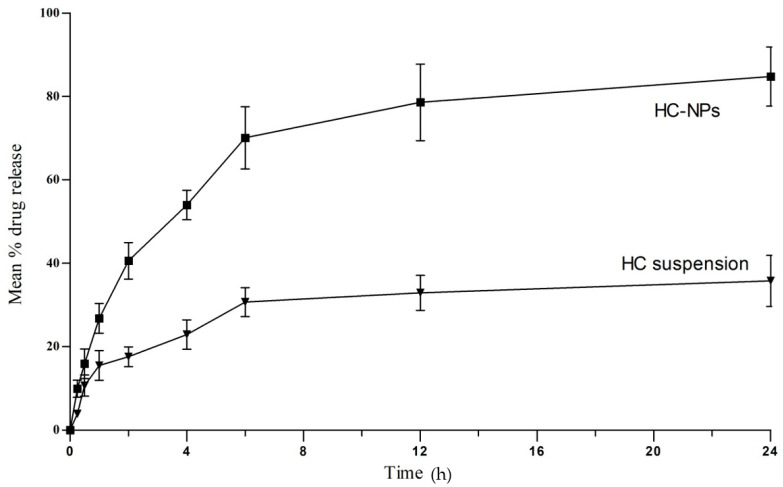
In vitro drug release of HC-NPs and suspension at pH7.4. HC-NPs showed statistically significant difference in % drug release (*p* < 0.05). Number of observations used in the test n = 6.

The drug release from HC-NPs and extract suspension at 37 ± 0.5 °C under test conditions showed low drug release 31.47 ± 1.27% (at 5 h) from suspension as compared to HC-NPs which showed higher release up to 83.09 ± 2.07% over 24 h, Figure 3.

The zero order, first order, Higuchi, and Korsmeyer–Peppas models were fitted to the in vitro release data. The highest correlation coefficient (R^2^) was preferred for selecting the order of release [32]. The correlation coefficient in optimised HC-NPs was found for the Higuchi model (R^2^ = 0.8379), followed by the first order (R^2^ = 0.7709) and the zero order (R^2^ = 0.6304) model which showed a mixed mechanism of release of Higuchi and first order, i.e., dissolution and diffusion control. The release pattern was examined by fitting the value in the Peppas model (R^2^ = 0.9289, n = 0.50) indicates that the release of drugs from the HC-NPs follows Fickian diffusion [33]. The drug release from degradable polymers can be driven by one of three mechanisms: (1) surface erosion of the polymer matrix, (2) cleavage of polymer bonds at the surface or inside the matrix’s bulk, or (3) diffusion of the physically entrapped drug. However, drug release is frequently the result of a combination of all three [34].

#### 2.2.7. Storage Stability Study of HC-NPs

The parameters did not exhibit any notable changes at accelerated conditions over the course of 90 days, as shown in Table 1.

### 2.3. Evaluation of HC-NPs Pluronic Gel (HC-NpsPO)

#### 2.3.1. Texture Analysis and pH

The textural properties of HC-NpsPO were analysed, and measures of cohesiveness, consistency, spreadability and viscosity were made (n = 3). The nanogel’s texture was described as a homogeneous light brown transparent gel with cohesiveness values of −98.93 gm, and consistency values of 183.34 ± 13.86 gm.sec, spreadability (spreading area, without putting weights) was 20.64 ± 0.75 cm^2^. And the viscosity value was 8387.45 ± 23.97, 7825.98 ± 230.67, 6661.67 ± 237.22 and 5726.32 ± 257.10. Centipoise was at shear rates of 60, 80, 100 and 120 s^−1^, respectively. HC-NPsPO showed a decrease in viscosity with increasing shear rates indicating pseudoplastic behaviour of the gel formulations (Supplementary files). Such behaviour is a desirable feature for topical preparations, as they should remain thin during application and thick at storage. The pH of HC-NPsPO was found to be 6.51.

#### 2.3.2. Drug Permeation through Excised Rat Skin

Cumulative drug permeation from HC extract pluronic gel (HCPO) was only 37.63 ± 2.74%, flux of 2.78 μg/cm^2^/h. compared to optimised HC-NPsPO (71.85 ± 7.57%) at 12 h with flux value 5.24 μg/cm^2^/h.

#### 2.3.3. Dermatokinetics Modelling of Topical Gel

**Figure 4:**
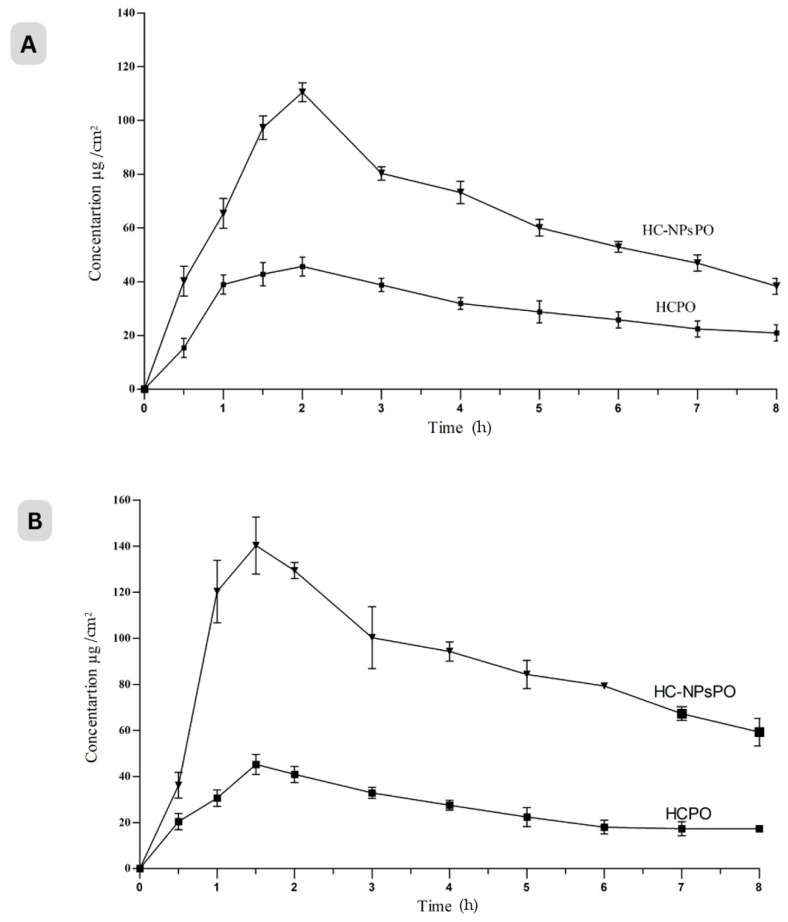
Amount of drug present in (**A**) the dermis and (**B**) the epidermis of rat at various time points. HC-NPsPO shows a significant difference in concentration as compared to HCPO (*p* < 0.05) in the dermis and epidermis, data are represented as mean ± SD, No of observation n = 6.

Figure 4 shows the drug distribution in the rat skin’s dermis and epidermis after being exposed to HC-NPsPO and HCPO at various time points. Table 2 displays the results of a one-way ANOVA on the numerical data of Cmax skin, Tmax skin, AUC 0–8 h, t1/2, skin disposal rate steady (Ke), and MRT.

### 2.4. In Vivo Analysis

#### 2.4.1. Acute Dermal Toxicity Study

The HC-NPsPO was safe for acute cutaneous toxicity up to a dosage of 2000 mg/kg, and treated animals’ fur, eyes, or behaviour did not change. There were no signs of skin irritation or an adverse reaction. Animals were healthy and active without mortality. Based on this, the formulation was accepted as safe for dermatological use.

#### 2.4.2. Skin Irritation Study

The results obtained for erythema and edema in order to measure the skin irritation of control, formalin and HC-NPsPO groups are shown in Table 3. The primary irritation index (PII) was calculated by adding the scores for erythema and edema. The substances that produce scores of two or less are classified as nonskin irritants [35]. As the score was found to be less than two, the findings on cutaneous irritation confirm that the formulation is not irritating. Hence, the prepared HC-NPsPOs have no significant skin irritation [36].

#### 2.4.3. Anti-Psoriatic Activity

**Figure 5:**
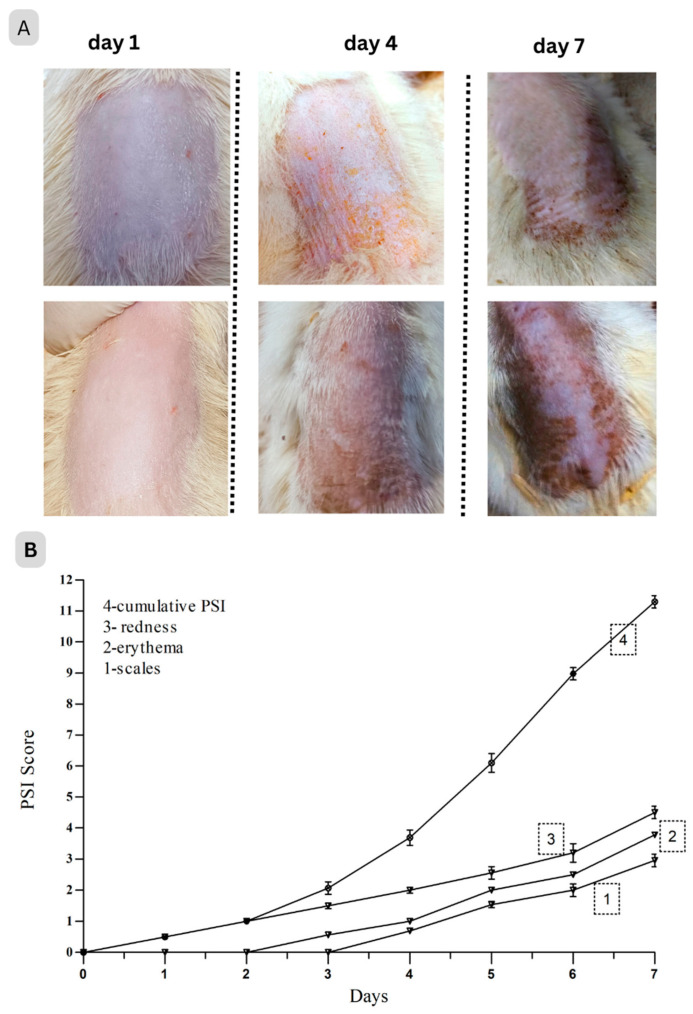
(**A**) Appearance of psoriasis-like skin inflammation on rat skin on different days after topical application of IMQ. Both images of each day represent two different rats of the same group. (**B**) Imiquimod-induced phenotypic changes (score 0–4) in rats and PSI.

IMQ was a strong immune stimulator [37]. On rat ears or dorsal skin, a high dose of IMQ (80 mg of 5% cream) applied topically several times over the course of seven days could cause erythema, scaling, keratinocyte proliferation, as well as an increase in the expression of immune cells and their associated cytokines, all of which are characteristics of psoriatic conditions [37]. The rat’s back skin began to show indications of erythema, scaling, and thickening after 3–4 days. In the exposed skin, various changes like redness, erythema, and silvery scales were noted visually and found to be increasing up to day 7, and the cumulative score (PSI) surged as shown in Figure 5 [38].

After day 7, treatment was started from day 8 onward for up to 2 weeks and the severity of psoriatic lesions was assessed. Skin lesions in the treatment groups significantly improved in terms of scaling, thickness, and erythema as well as PSI (Table 4). The results showed that HC-NPsPO markedly alleviated the clinical symptoms of psoriasis from rat skin provoked by IMQ.

#### 2.4.4. Histopathological Study

**Figure 6:**
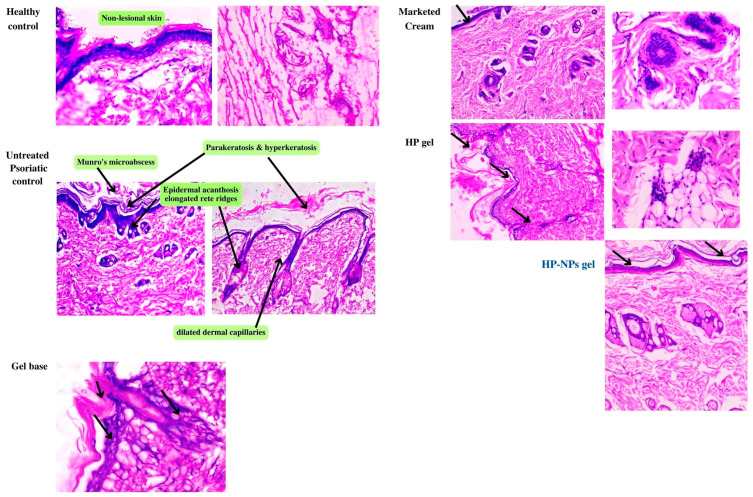
A Histopathological changes observed in H&E stained sections showing changes in rat skin. Healthy control shows the non-lesional epidermis. Untreated psoriatic control group skin shows epidermal acanthosis with elongated rete ridges, munro’s microabscess & dilated dermal capillaries. The gel base group has no improvement in psoriatic lesions. Marketed group shows hyperkeratosis, dilated blood capillaries, reduced epidermal acanthosis& rete ridges, and overall cytoarchitecture of skin is improved. HP gel group had no marked reduction in psoriatic lesions. HP-NPs gel group exhibited normal structure of the epidermis and underlying dermis with skin appendages. Magnification 400 px, scale bar 75 μm. Black arrow indicates reduced epidermal acanthosis& rete ridges.

**Figure 7:**
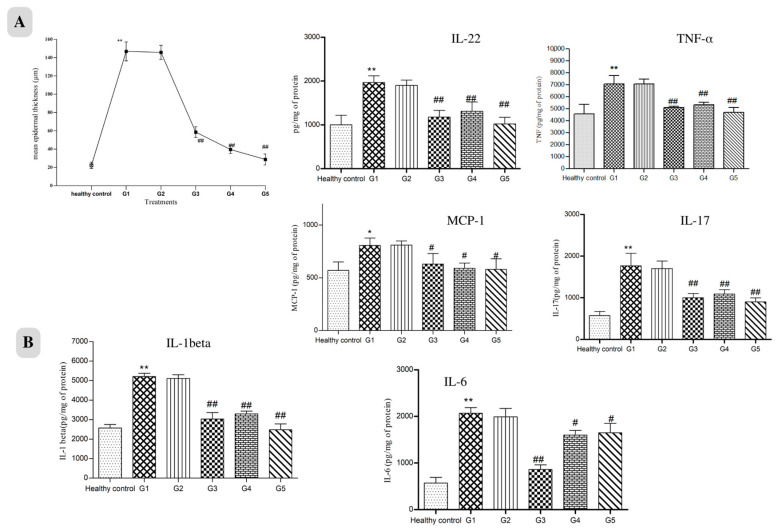
(**A**). Epidermal thickness of different treatment groups after 21 days. Data are represented as mean ± SD, n = 6, * *p* < 0.5,** *p* < 0.01 vs. healthy control, ## *p* < 0.01 vs. untreated control. (**B**). Effect of treatments on pro-inflammatory cytokines and vascular endothelial factors in the dorsal skin of IMQ induced psoriasis in rats. Values are mean ± SD (n = 6). Where ** *p* < 0.01 vs. healthy control, # *p* < 0.05, ## *p* < 0.01 vs.G1 group G1-Untreated control, G2-Gel base, G3-Marketed preparation, G4-HCPO, G5-HC-NPsPO.

Hyperkeratosis, Inflammatory cell infiltration, increased epithelial thickness (acanthosis) with clubbed rete ridges, Munro’s microabscess and dilated dermal capillaries were seen in the untreated psoriatic control group (Figure 6). Healthy control shows the non-lesioned epidermis. Additionally, in the gel-treated group, all signs of psoriatic lesions were observed. The marketed group shows hyperkeratosis, narrowed blood capillaries, reduced epidermal acanthosis and rete ridges, and the overall cytoarchitecture of skin is improved. The HCPO group had no marked reduction in psoriatic lesions. The epidermis and underlying dermis, along with skin appendages, showed healthy structure in the HC-NPsPO group. The epidermal thickness of the treated animals showed a remarkable decrease in thickness compared with that of untreated animals, as shown in Figure 7A.

#### 2.4.5. Downregulation of Inflammatory Cytokines

The levels of critical inflammatory mediators (IL-17, IL-22, IL-6, IL-1β, TNF-α, and monocyte chemotactic protein 1 (MCP-1/CCL2)) in psoriatic skin tissues were all significantly raised following IMQ use, according to ELISA results. These pro-inflammatory cytokines were downregulated following topical treatment of marketed and prepared nanogel, as shown in Figure 7B. Our results showed that HC-NPsPO reduced inflammatory cytokine levels towards normal, which is one contributing factor towards effective psoriasis management [15].

## 3. Discussion

An experimental design was used to optimise HC-NPs, choosing the liquid and solid lipid contents to produce the particle size (86.11 nm), drug content (89.79 ± 2.39) and maximum entrapment efficiency (73.05 ± 0.98%). The efficiency of nanophytosomes for drug delivery depends on the control of their size and zeta potential. The magnitude of the zeta potential (−10.40 mv) reflects the stability of the formulation, whereas the zeta potential’s sign reveals whether positive or negative charges predominate at the surface [39].

The DSC study indicated an encapsulation of the drug within the carrier. A single crystal of a nanostructure may be focused upon via TEM, allowing for the clarification of its precise crystalline geometry. According to TEM images, HC-NPs were smooth, and their size corresponded well to zeta sizer results. The topography obtained from this imaging suggested that the thin film hydration method produced NPs with a homogeneous size distribution, and crystalline and porous character [28].

These nanostructures were able to provide a good entrapment value despite their lipophilic character. Phospholipids serve as precursors in the encapsulation process and assure the optimal stability of nanostructures [40]. Our approach indicates sustained release of entrapped drugs over a period of 24 h, which is comparable to the release pattern of drugs from nano-deliveries as documented in earlier research [41,42]. In HC suspension (without any excipients), the drug is released by dissolution. Total flavonoids (Quercetin equivalent) were considered active, so here the drug release means the release of quercetin present in the extract. It is hydrophobic in nature, and therefore the release of free quercetin (extract suspension) is lower than that of nanoparticles (HC-NPs) [43,44]. In vitro drug release was dependent on the particle size, the morphology, and the crystallinity of the drug. Both physical and chemical changes were observed during the stability study of 90 days. Physical stability was analysed in terms of physical appearance and particle size, whereas chemical stability depended on drug content and entrapment efficiency. The results showed that the stability characteristics of the HC-NPs did not change significantly over the period of the study.

The high cohesiveness of prepared nanogel makes it suitable for topical application. For the gel to stay in the skin for a longer period of time, it is also preferred that it be non-irritating and has high spreadability [28], which is shown by HC-NPsPO. The pH of the topical formulation is an important determinant, particularly in the case of psoriasis. The pH of the cell and extracellular area is normally almost neutral (pH 7.2); nevertheless, psoriasis plaque is distinguished by a slightly acidic pH [45]. Consequently, the pH value of HC-NPsPO, which was 6.52, fell within the permissible range for topical treatment in cases of psoriasis [33]. Overall, prepared HC-NPsPOs have the characteristics desired for dermal delivery.

The incorporation of HC-NPs into the gel may be the cause of the notable shift in the percentage of drug transport. In addition to providing stability to the formulation, lipids and surfactants of NPs increase the solubility of the drug, which increases its penetration. The gel’s nanosize enables it to permeate the skin’s inherent layers and increases the absorption of drugs. It shows that drugs remain in the skin layers for an extended time, which is valuable for the effective management of psoriasis [46]. Leciva-S90, which is present in HC-NPs, affects the fluidity of the membrane to help the drug pass through the pores [33]. The improved HC-NPsPO penetration was likely caused by the gel’s nano size and the emergence of an occlusive layer on the skin.

The ability of NPs to increase partitioning across the skin lipid bilayers may account for the highest retention of HC-NPsPO in the epidermis and dermis layers. In the epidermis, the T skin maxes of the HC-NPsPO and HCPO were likely similar. From the findings, the concentration of a drug is measurable following 30 min of topical application. HC-NPsPO delivery showed rapid absorption of the drug, reaching the Cmax within 1.6 h in the epidermis and 3 h in the dermis, following topical application. The AUC of HC-NPsPO was also significantly greater in the dermis as compared to the epidermis. More drugs in skin layers and higher Tmax obtained from HC-NPsPO supported enhanced permeation and retention in comparison to HCPO. Prepared HC-NPsPO is expected to embed in the superficial layers (epidermis) and the drug diffuses into deeper layers (dermis). The average time that molecules of a dosed drug spend in the skin layers was higher in HC-NPsPO than in HCPO. The results showed that the developed nanogel was more effective in reaching the deep layers of the skin for psoriasis treatment. The drug concentration in HCPO was seen to decline with time until the 8th hour of the experiment; however, the drug concentration in HC-NPsPO was found to be quantifiable. The above findings of the HC-NPsPO delivery suggest increased penetration of the drug when applied topically, which is in line with documented scientific data [33,46,47].

Psoriasis is a type of skin disease that is prevalent, persistent, and recurrent. Psoriasis characteristics were replicated in rat models by using IMQ. On days 2–4 after starting IMQ application, the rat’s dorsal skin appears thickened and erythemic with scaling. There were visible signs of inflammation which gradually grew in intensity up to days 5–7. In the HC-NPsPO-treated group, it was observed that there was a decrease in all the visible signs, like swelling and redness. There was no significant change in all parameters for the untreated control group, and the data are tabulated in Table 4. The group treated with marketed cream also demonstrated a decline in the psoriatic parameters. So, with the observation obtained from the scoring of all groups, we could conclude that the HC-NPsPO was more promising as compared to the marketed one in terms of efficacy. Additionally, all groups were evaluated for cytoarchitectural changes by histopathology. When IMQ-induced rat skin was compared to healthy control skin, histological analysis revealed increased epidermal thickness, hyperproliferation of keratinocytes, granulocyte infiltration, the presence of microabscesses, and capillary loop dilatation. The main clinical and histological differences between psoriatic skin and other inflammatory skin diseases are changes in epidermal architecture and variations in keratinocyte differentiation [48,49]. Histological abnormalities were significantly reduced in animals treated with HC-NPsPO. In the healthy control skin, intact epidermis and viable dermis can be seen. The HC-NPsPO-treated skin almost returns the altered skin architecture to normal. The untreated control had visible psoriatic lesions in the epidermis and dermis.

The majority of the chemokines implicated in the onset of psoriasis are released by keratinocytes. Antimicrobial peptides and proinflammatory cytokines are also produced by activated keratinocytes, and they all together increase inflammation. The data acquired from the results showed an increase in cytokine expression after IMQ application and confirmed the pathogenesis of psoriasis [50]. Inflammatory cytokines were measured in the skin samples of healthy control, untreated control, gel base group, marketed gel group, HCPO- and HC-NPsPO-treated rats by ELISA assay. When skin samples from IMQ-treated rats were compared to healthy controls, the expression of inflammatory mediators was found to be considerably higher. Meanwhile, marketed and developed nanogel resulted in a significant decrease in the expression of all six evaluated cytokines (IL-17, IL-22, IL-6, IL-1β, TNF-α, and MCP-1/CCL2). The treatment of rats with HCPO also resulted in a decrease in cytokine level but a marked decrease by the HC-NPsPO suggests the protective effect against IMQ-induced inflammatory lesions in rats. Attenuated MCP-1 production leads to diminished infiltration of monocytes into the inflamed tissue. Purzycka-Bohdan et al. also highlighted that anti-TNFα therapy reduced the expression of CCL2/MCP-1 within psoriatic skin robustly; however, it only moderately decreased CCL2/MCP-1 plasma levels [51].

## 4. Materials and Methods

### 4.1. Reagents and Drugs

The reagents and drugs used in this study were: Dried hydroalcoholic (1:1 methanol/water) extract of *Hedyotis Corymbosa* leaves (Ambe NS Agro Products Pvt. Ltd., New Delhi, India, a WHO GMP, USFDA certified organic and botanical extract producer), Leciva-S90 (purified soy lecithin with minimum 90% phosphatidylcholine); VAV life sciences, Mumbai, India, Pluronic^®^F-127 (Poloxamer 407); Sigma Aldrich, Bangalore, India, Quercetin hydrate; Tokyo Chemical Industry (India) Pvt. Ltd. Hyderabad, Tween^®^80 (Polysorbate 80); Loba Chemie of Bombay, India. Pectin (polysaccharide) and gallic acid (3,4,5-trihydroxybenzoic acid) from GLR Innovations, New Delhi, India.

### 4.2. Characterisation of Hedyotis Corymbosa extract (HCE)

The following test parameters were disclosed in the manufacturer’s certificate of analysis (*Hedyotis Corymbosa* extract, Report no. AMB/COA/2022, Batch no. AMBHC2223): light brown colour powder with characteristic odour and pH (1% aqueous solution) 5–9, flavonoids content by UV method on dried basis was reported as 10.02% *w*/*w*. The assay (UV-Vis on a dry basis) revealed polyphenols 30% and total ash as not more than 5%. The particle size of the extract powder ranges from 395 to 425 µm. Total heavy metals (less than 10 ppm) were recorded as Lead (3 ppm), As (1 ppm), Cd (1 ppm), and Hg (0 ppm) (0.5 ppm). The lack of *E. coli*, *Salmonella*, *Pseudomonas aeruginosa*, *Staphylococcus aureus*, and yeast counts was revealed by a microbiological analysis (cfu/gm) of the extract.

Preliminary phytochemicals tests were performed as per previously reported literature [52], HC leaf extract showed the presence of proteins, carbohydrates, phenols, tannins, flavanoids, saponins, steroids, terpenoids, glycosides and other polysaccharides.

#### 4.2.1. Total Flavonoid and Phenolic Content Estimation of HCE

The total flavonoid concentration of the extracts was measured using a colourimetric technique based on aluminium chloride [53], using a quercetin standard curve, and the findings are given as mg QeE/g Extract DW. The calibration curve of quercetin standard solutions in methanol was prepared at 376.0 nm using UV spectrophotometer (Shimadzu UV-1800, Shimadzu Analytical, Mumbai, India) covering the concentration range of 0.2 to 1.0 mg/mL, r^2^ = 0.9675. Stock solution (1 mg/mL) was prepared by dissolving quercetin in methanol; subsequently, the desired concentration range was prepared by appropriate dilution of stock.

The Folin–Ciocalteu technique was used to determine the total phenolic content of the extracts [54]. Absorbance was measured (735 nm) using a spectrophotometer (Shimadzu UV-1800, Shimadzu Analytical, Mumbai, India), and the findings were represented as gallic acid equivalents from a gallic acid reference [54]. Calibration curve concentration range was 10–80 µg/mL, r^2^ = 0.8972.

#### 4.2.2. Polysaccharides Quantification

In 96-well microplates, 150 mL concentrated sulfuric acid was swiftly added to 50 mL extract solution (5%). After 15 min of shaking, 30 mL of 5% phenol in water was mixed [55]. After incubation for 5 min at 90 °C in a water bath, the plate was cooled. The absorbance at 492 nm was measured using a microplate reader (SPECTROstar Nano, BMG Labtech, Mumbai, India). After testing each sample in five wells, average was computed. Using a pectin calibration curve, the data were represented as pectin equivalents [55].

### 4.3. Preparation and Optimisation of Nano-Phytosome of HCE

#### 4.3.1. Excipient Screening and Compatibility

The choice of solid lipid was fixed on the estimated solubility of the extract in order to produce a visible clear solution in lipid when viewed from the naked eye. In a volumetric flask, extract (10 mg) and different amounts of chosen lipids, Leciva-S90 and Precirol^®^ ATO 5 (Glyceryl distearate, as a gift sample from Gattefosse, Mumbai, India), were dissolved in ethanol. In normal light, the extract’s estimated solubility was determined after dissolving the lipids in a volumetric flask [46,56]. Since the extract was more soluble in Leciva-S90, it was used to prepare a nanophytosome. HCE-estimated solubility in ethanol, methanol, hexane, chloroform, polyethylene glycol, and water was tested using USP rules and found to be soluble in all.

Due to its low toxicity, compatibility, stability, formulation clarity, and pH independence, a nonionic surfactant was selected. Tween 80 was added as a surfactant due to its miscibility with the extract. Based on the outcomes of preliminary batches, the ratio of surfactants that generated stable and smaller size NPs was chosen.

#### 4.3.2. Preparation of HC Loaded Nano-Phytosome (HC-NPs)

To prepare NPs, a previously published thin film hydration process was used with some modification [30]. Leciva-S90, Tween 80, and the extract were dissolved in a binary mixture of the organic solvent’s ethanol and chloroform (2:1). Subsequentially, mixture was homogenised then transferred to a round bottom flask and placed on a rotary evaporator (Heidolph Laborota 4000 Series, Heizbad, Germany) at 100 rpm under reduced pressure at 60 °C, where a dry thin coating formed on the inner flask wall. Film was hydrated further with phosphate buffer pH 7.4 at 50 °C (5–10 °C above the amphiphiles phase transition temperature) [57,58]. After being kept at room temperature for 4 h, it was sonicated for specified time, as per design, to shatter the multilamellar phytosomes [30].

#### 4.3.3. Experimental Design

Using Design-Expert v13 (version 13.0.13), the Box–Behnken design was used to examine the effects of independent factors on the properties of HC-NPs. In a response surface, randomised Box–Behnken design, the independent variables phospholipid concentration, Tween 80, hydration time, and sonication time were modified at three levels for the optimisation. The values of three coded levels of four components were presumed after preliminary batch testing, and they are shown in Table 5. Table 5 presents all independent variables and their outcomes [59]. The amount of the drug was kept constant at 100 mg.

Considering the proximity of the desire factor to 1, the Stat Graphics Centurion program’s numerical point prediction was used to choose an optimum NP formulation. Desirability function (0.778) was probed to acquire an optimised formulation [59]. Table 6 lists the prerequisites for optimum formulation as anticipated by the programme. Further preparation and characterisation of optimised HC-NPs were performed.

#### 4.3.4. Structural Evaluations and Characterisation of HC-NPs

##### Differential Scanning Calorimetry (DSC)

Thermal response of the samples was carried out using DSC (DSC 4000, Perkin Elmer, Mumbai, India). Sample containing sealed pans were heated from 50 to 350 °C at a rate of 20 °C/min to obtain DSC spectra. The obtained spectra were compared [28].

##### Particle Size (PS), Polydispersity Index (PDI) and Zeta Potential

Dynamic light scattering approach at a fixed angle of 90° and 25 ± 1 °C temperature was used to determine PS and PDI of HC-NPs using particle size analyzer (Litesizer 100, Anton Paar, Gurugram, Haryana, India). Zeta potential examinations were carried out by Malvern Zetasizer. Samples were assessed in triplicate [30].

##### Transmission Electron Microscopy (TEM) of HC-NPs

The morphology of the samples was evaluated using a TEM (Model-JEM2100F, Japan Electron Optics Laboratory (JEOL), Tokyo, Japan) at 80 K. Sample was mounted on carbon grids and negatively stained with phosphotungestic acid (2% *w*/*v*) [30].

##### Entrapment Efficiency (% EE)

The EE% of HC-NPs were measured by the indirect method using cool centrifugation technique [30]. Prepared HC-NP suspension (10 mL) filled in centrifuge tube and centrifugation was performed at 20,000 × *g* at 4 °C for 30 min to separate the free drug. The collected supernatant was filtered and diluted with phosphate buffer pH 7.4 and the drug (quercetin) concentration was determined by UV spectrophotometer at 376 nm using quercetin standard curve. Blank NPs with the same dilution were used in a reference cuvette. The EE% will be calculated using the following equation [60].

$$EE\left(\%\right)=\frac{Total\;drug-Free\;drug}{Total\;drug}\times 100$$

where the total drug is the amount of drug added, while the free drug is the free amount of drug present in the supernatant solution.

##### Drug Content

A total of 10 mL of phosphate buffer pH 7.4 and 1 mL of HC-NPs were sonicated for 15 min to lyse. Following that, it was filtered through a 0.45 µm syringe filter and, diluted if required. Absorbance was measured at 376 nm by UV spectrophotometer (Shimadzu UV-1800, Shimadzu Analytical, Mumbai, India) and the amount of drug (quercetin) was calculated from quercetin standard curve [60]. Drug content was determined by the formula:

$$Drug\;content\;\left(\%\right)=\frac{Amount\;of\;drug\;obtained\;}{Amount\;of\;drug\;added}\times 100$$

##### In Vitro Drug Release

The drug release profiles from HC-NPs and extract suspension were obtained by using a dialysis bag (molecular weight: 12–14 kDa pore size: 2.4 nm, Himedia) after soaking in distilled water for 12 h. A known amount of HC-NP dispersions was kept in a dialysis tube and subjected to the receptor fluid (phosphate buffer, pH 7.4), stirred at 100 rpm and kept at 37 °C in a shaking water bath. Aliquots were withdrawn at pre-determined time intervals at 0, 0.25, 0.5, 1, 2, 4, 6, 12 and 24 h. The volume of receptor fluid was maintained constant by refilling the same medium after each withdrawal to conserve the sink condition. The amount of drug (quercetin present in the extract) released in the medium was measured by UV after necessary dilutions. Dimensions of the dialysis bag were kept constant for all the experiments. Analysis was performed in triplicate [28].

The release data were fitted into several kinetic model (zero order, first order, Higuchi, and Korsmeyer–Peppas) equations to find the possible mode of the drug release from NPs. For zero order dissolution (cumulative drug release), data were plotted between % cumulative drug release vs. time, first order kinetics log % cumulative drug release vs. time, for Higuchi graph between % cumulative drug release vs. root time. Korsmeyer–Peppas graph was used, typically plotting log % cumulative drug release as a function of log time. The regression equations were calculated and the correlation coefficients were determined [61].

Zero and first order models are dissolution-controlled whereas Higuchi model is based on diffusion-controlled drug release. Korsmeyer–Peppas is to ensure Fickian (diffusion exponent n ≈ 0.45) or non-Fickian drug diffusion (diffusion exponent 0.45 < n < 0.89) from drug–polymer matrix and reservoir system. Diffusion that takes place in solids and polymers is very different from liquid and gases. Fick’s laws are a set of equations that can be used to explain the steady-state (time-independent) diffusion in solids. Fickian diffusion sets no boundaries, and therefore the drug is diffused directly from the formulation to the media. On the other hand, non-Fickian forms of diffusion have sharp boundaries like dry, glassy and swollen state of polymer used in formulation, also known as anomalous diffusion [62].

##### Physical Stability of HC-NPs

HC-NPs were packed in a sealed vial and stored in a stability chamber (Humidity chamber, Kesar Control System, Gujrat, India) at 40 ± 2 °C/75 ± 5% RH storage condition, according to the International Conference on Harmonization, Q1A(R2) [63,64]. Samples were removed periodically and physical appearance, particle size, drug content, and % EE were analysed. [57,58].

#### 4.3.5. Incorporation of HC-NPs in Pluronic Gel and Characterisation

Since HC-NPs detach easily from the skin, they were embedded into a gel base [46]. Pluronic F127 (15% *w*/*v*) dispersion in double distilled water along with 0.1% of chlorocresol (as preservative) were used as gel base. Finally, NPs equivalent to 100 mg of extract [28,38,65] were added into this preformed gel base with constant agitation to obtain a homogeneous 10% *w*/*w* HC-NPs pluronic gel (HC-NPsPO) [33]. Similarly, conventional HC extract pluronic gel (10% *w*/*w* HCPO) was prepared.

##### Estimation of pH and Texture Profile Analysis of the HC-NPsPO

A pH meter (Accumet AE150 Benchtop pH meter, Fisher Scientific, Mumbai, India) was used to assess the pH of the prepared HC-NPsPO.

The texture properties of formulation are an important parameter in optimisation of topical gel. These properties will affect applicability of the formulation at the administration site and therapy outcome [66]. We performed texture analysis after incorporating nanophytosome into the pluronic gel base. A software-controlled texture analyzer (Brookfield CT3-1000 Texture Analyzer with Texture Pro CT software 1.0) was used to determine the gel’s texture. In order to prevent the formation of air bubbles within and to provide a flat surface, 50 g of the nanogel was added to a 100 mL beaker and a 40 mm-extrusion disc was placed at the centre, over the test container. Care was taken to hold the container firmly in place to prevent it from lifting when the probe returned to start position [66]. The disk was inserted into the deepest part, where active surface was reported, i.e., the point at which the bottom surface of the disc comes into contact with the product. At this point, the probe moved back to its real position when maximum force was applied. An output graph was typically plotting resisting force (N) as a function of time [66]. The gel’s mechanical characteristics, including consistency, cohesiveness, spreadability, and viscosity were measured [46]. All tests were conducted at room temperature (25 ± 2 °C).

Cohesiveness is the tendency of a product to cohere or stick together it is defined as the work required to deform the gel in the down movement of the probe. Which is obtained from the texture analysis output graph (the negative area under the force–time curve). The consistency of the gel showed that the higher the area of curve in the output graph (the positive area up to maximum value under the force–time curve), the higher the resistance to withdraw the gel formulation [67]. A texture analyzer with a specialised spreadibility test fixture was used to quantify spreadibility. A cone-shaped probe is connected to the analyzer and aligned with a cone-shaped receptacle on the base beneath the probe. The sample is kept in this receptacle. The probe subsequently goes downward at a predetermined speed to compress. The probe quantifies the amount of force required to penetrate the gel’s surface as it moves into the sample holder [68]. Spreadibility of HCNPsPO was checked to verify the ease of application and its capacity to spread over a wider area.

Viscosity measurements were carried out at 25 ± 0.1 °C temperature using a rheometer (Brookfield rheometer DV-II+ Pro, Mumbai, India). Briefly, the gel samples were allowed to rest for 5 min prior to analysis. Lower the viscometer into the gel until the surface of gel is level with the groove mark on the viscometer spindle (LV-3(63) was selected on trial–error basis). The shear stress vs. shear rate curve with a shear rate range of 60–120 s^−1^ was obtained. It was used to determine viscosities (Supplementary file). Viscosities are measured at different shear rates of 60, 80, 100 and 120 s^−1^ to determine the flow behavior of gel.

##### Ex Vivo Skin Permeation Study

The study was examined through Franz diffusion cell. The rat skin was removed, cleared of adhering fat layers, and mounted on Franz diffusion cells with a cross-sectional area of 1.77 cm^2^ and receptor volume of 9.0 mL. To examine the skin’s integrity, a methylene blue dye test was conducted [69]. Phosphate buffer pH7.4 was filled in receptor compartment and constantly stirred (100 rpm) at 37 ± 1 °C throughout the experiment using a magnetic stirrer [33]. The receptor compartment was bubbled with air to provide oxygenation and agitation. Then, 1 gm of the HC-NPsPO was applied to the pre-incubated (20 min) skin. At predetermined time points (0, 0.25, 0.5, 1, 2, 4, 6, and 12 h), 1 mL sample was withdrawn from the receiver and replaced with an equivalent volume of buffer to maintain the sink condition. UV-vis spectroscopy was used for sample analysis. The same procedure was carried out again for HCPO.

##### Ex Vivo Dermatokinetics Study

In order to carry out a dermatokinetics evaluation, HC-NPsPO levels in the epidermis and dermis were measured at various time points. The dermatokinetics test was used to quantify the amount of drug present in different layers of excised rat skin [70]. The content of drugs in epidermis and dermis layer of dorsal skin at different times was estimated by putting the HC-NPsPO and HCPO to rat skin mounted in Franz diffusion cells as stated in ex vivo skin permeation study [47]. In this study, however, the complete skin was taken from the Franz diffusion cell at various times; 0, 1, 2, 4, 6 and 8 h [46,71]. The skin was cleansed with pH 7.4 buffer to remove any adherent gel before being placed in warm water at 60 °C for 2–3 min to aid in the separation of epidermis and dermis.

The resulting wedge of the rat skin, showing a split of epidermis and dermis, was pulled out and separated using forceps [71]. The removed skin layers were crushed and immersed in 5 mL of ethanol for 24 h to extract the drug. A membrane (0.45 μm) was used to filter the fluid and the filtrate was analysed for drug concentration using UV-VIS spectroscopy as previously described. The drug content per cm^2^ of skin was plotted against time for the epidermis and dermis. Dermatokinetic T_skinmax_, C_skinmax_, AUC_0–8 h_, t1/2, skin disposal rate steady (Ke), and MRT were assessed [33,71] for non-compartmental pharmacokinetic model.

#### 4.3.6. In Vivo study

##### Animals and Ethical Statement

The study used healthy female Wistar Albino rats weighing an average of 191 g [72] purchased from veterinary house, All India Institute of Medical Sciences (AIIMS, New Delhi, India). Rats were acclimatised in a room temperature and humidity environment with a 12-hour light/dark cycle and free access to food (AIN-93 Rodent Diet) and water. The Ethics Committees of I.T.S. College of Pharmacy, Ghaziabad, India, accepted all experiment protocols (Protocol no. ITS/06/IAEC/2022). To eliminate confounding factors, all animals in this study were of the same group size, strain, sex, and age [73]. To prevent unavoidable pain, appropriate animal housing and husbandry practices were followed throughout the experiment [74]. This single-center, open-labelled study lasted for 21 days. Sample size calculations were based on previously published research, retrospective data on potential loss during follow-up, and intermediate data. The estimated sample size (total of 36 animals divided into 6 groups and in each group n = 6) is our logical guess, to make study findings valid statistically.

##### Acute Dermal Toxicity

The OECD guidelines No. 402 were used to determine the acute dermal toxicity in female Wistar Albino rats. Three groups of three animals each were formed from the nine animals [75]. Depilatory product (VEET hair removal cream) was used to remove 10% of the test animals’ body hair from their dorsal region 24 h prior to the test. Group I animals were used as a control, Group II animals received 1000 mg/kg of HC-NPsPO topically, and Group III animals received 2000 mg/kg [76]. Animals received a single application of the HC-NPsPO on the first day of the experiment, and their fur, eyes, behaviour, and toxic dermal responses were monitored over the next 14 days.

##### In Vivo Skin Irritation Test

The Draize skin test scores were assessed for the skin irritation potential [46]. A comparative examination was carried out on rat shaved dorsal skin to test the irritating potential of the HC-NPsPO, with a standard irritant as a positive control (Formalin solution, 0.8% *v*/*v*) and no treatment as a negative control. After 24 h, animals were routinely evaluated for symptoms of erythema and edema, and severity ratings ranging from 0 to 4 were assigned [35].

##### Establishment of Psoriasis-Like Dermatitis Rat Model and Treatment Protocol

To create a rat model of psoriasis, 80 mg of commercial 5% *w*/*w* IMQ cream (Imiquad cream, Glenmark, India) was topically applied twice daily (morning and evening) for seven days in a row to induce a psoriasis-like dermatitis [15,50]. An objective grading system was developed using the psoriasis area and severity index (PASI). Redness, erythema, and scaling were all scored separately on a scale of 0 to 4: 0, none; 1, low; 2, moderate; 3, marked; and 4, extremely marked [77]. The psoriasis severity index (PSI scale 0–12) was measured by the cumulative score (sum of redness, erythema, and scaling) [18]. Randomisation is a well-accepted approach for reducing bias or confounders that should be taken into account while designing an experiment.

Following psoriasis induction, rats were randomly assigned into six groups, each including six animals: G1 (untreated psoriatic control), G2 (gel base group), G3 (Giosun psoriasis cream, Giosun Healthcare Pvt. Ltd. India), G4 (HCPO-treated), G5 (HC-NPsPO), and healthy control groups. Except for the healthy control, the respective treatment was begun on day 8 and applied twice daily for 21 days [38]. Gel base (80 mg/cm^2^), marketed gel (80 mg/cm^2^), HCPO (80 mg/cm^2^), and HC-NPsPO (80 mg/cm^2^) were all applied twice a day on psoriatic skin [46]. Rats in the untreated control group applied petroleum jelly according to the same schedule. In the end, the animals were euthanised with thiopentone sodium (150 mg/kg) and skin samples were preserved in 10% formalin [4]

##### Histopathology

Skin samples were formalin-fixed, paraffin-embedded, sectioned into 5-µm thick sections, stained with haematoxylin and eosin (H&E), and viewed under a light microscope (OLYMPUS BX60, 200 magnifications). Image analysis software (OLYMPUS DP72 Ver.5174) was used to determine the average optical density of related indicators, including acanthosis of skin [15,78].

##### Detection of Inflammatory Cytokines in Skin Tissues Lysate

Tissue Protein Extraction Reagent (Thermo Fisher Scientific) was used to extract protein from crushed skin samples. For 20 min, the proteins were centrifuged at 12,000× *g*. The supernatant was then collected, and the protein contents were determined using the Erba total protein assay kit. Commercially available Invitrogen/Thermo Fisher Scientific, enzyme-linked immunosorbent assay (sandwich ELISA) kits (Thermo Fisher Scientific India Pvt. Ltd., Powai, India) were used for IL-22 (IL-22 Mouse ABTS ELISA Development Kit), IL-6 (IL-6 Mouse ELISA Kit), IL-17 (IL-17A Mouse ELISA Development Kit ABTS), TNF-α (TNF alpha Mouse ELISA Kit, Elabscience) IL-1β (IL-1β Mouse ELISA Development Kit ABTS) and MCP-1/CCL2 (mouse MCP-1 ELISA kit, FineTest). All tests were performed as per the manufacturer’s instructions manual [15].

#### 4.3.7. Statistical Analysis

The experimental results were represented as mean standard deviation and were analysed using one-way ANOVA and the Tukey multiple comparisons test. For statistics, GraphPad Prism 5 (San Diego, CA, USA) was used. In all groups, *p* < 0.05 was considered statistically significant.

## 5. Conclusions

At the moment, traditional psoriasis therapeutic approaches have a lot of shortcomings such as limited effectiveness, itchy skin, immunosuppression, and so forth, which suggests the use of herbal alternatives with minimal adverse effects. This study emphasises the benefits of the developed HC-NPsPO as a viable topical administration system with improved penetration, drug deposition into deeper skin layers, and reduced systemic absorption when compared to extract, demonstrating the usefulness of innovative nanosizing techniques in boosting the biological activities of herbal extracts. Skin dermatokinetic results and preclinical data, as well as downregulation of expression of inflammatory mediators IL-17, IL-22, IL-6, IL-1β, TNF-α, and MCP-1, confirm the nanogel’s potentiated antipsoriatic efficacy with a positive safety profile. The score obtained from the skin irritation study revealed non-irritancy of the developed formulation. Hence, the obtained results indicate that developed HC-NPsPOs are potential carriers for the effective and safe topical delivery of herbal extract. Previously, the hepato-protective, anti-colorectal cancer, atopic dermatitis, and antimalarial potential of Hedyotis corymbosa were reported. In this study, we elucidated the antipsoriatic efficacy of the herb. Nevertheless, clinical perspective examinations are highly encouraged to validate its efficacy and give more insight as an alternative or adjuvant to the currently available therapies for autoimmune disorder psoriasis.

## Figures and Tables

**Table 1:** Stability study of HC-NPs at accelerated conditions as per ICH guidelines.

Storage	Physical Appearance		Particle Size (nm)		Drug Content (%)		EE (%)	
	45 Days	90 Days	45 days	90 Days	45 Days	90 Days	45 Days	90 Days
40 ± 2 °C/75 ± 5%	clear	clear	89.61	92.11	88.71	86.02	72.19	71.08

**Table 2:** Ex vivo dermatokinetic parameters (mean ± SD) of HCPO and HC-NPsPO in dermis and epidermis layer of rat dorsal skin, Number of observations n = 6.

Parameters	HCPO		HC-NPsPO	
	Epidermis	Dermis	Epidermis	Dermis
Tskin max (h)	1.5 ± 0.03	2 ± 0.02	1.6 ± 0.04	3 ± 0.03
Cskin max (µg/cm^2^)	68 ± 1.32	37 ± 1.23	196 ± 11.23	102 ± 7.25
AUC 0–8 (µg/cm^2^ h)	204 ± 12.32	241 ± 10.23	425 ± 14.29	792 ± 21.45
Ke (h − 1)	1 ± 0.01	1.55 ± 0.04	1.67 ± 0.04	1.8 ± 0.07
MRT	1.68 ± 0.03	2.0 ± 0.02	4.21 ± 0.06	4.46 ± 0.03

Tskin max, time to maximum concentration; Cskin max, maximum concentration; AUC, area under curve; Ke, elimination rate constant, MRT, mean residence time.

**Table 3:** Primary irritation index score of treated rats.

Time (h)	Control		Formalin (0.8%)		HC-NPsPO	
	Erythema	Edema	Erythema	Edema	Erythema	Edema
0	0	0	0	0	0	0
8	0	0	2	2	1	1
16	0	0	3	4	0	1
24	0	0	4	4	0	1
Mean Score ± SD	0	0	2.22 ± 0.16	2.5 ± 0.36	0.25 ± 0.012	0.69 ± 0.16
PII	0		4.72		1.00	

**Table 4:** Evaluation of PSI score (0–12) of different groups after treatment, data represented as mean ± SD, Number of observations = 6.

Days	Group 1 (Untreated Psoriatic Control)	Group 2 (Gel Base Group)	Group 3 (Giosun Psoriasis Cream)	Group 4 (HCPO-Treated)	Group 5 (HC-NPsPO-Treated)
7	10.89 ± 0.36	10.03 ± 0.35	6.58 ± 0.87	8.35 ± 0.05	7.02 ± 0.56
14	10.78 ± 0.47	10.45 ± 1.09	4.78 ± 0.46	7.89 ± 0.057	4.03 ± 0.25
21	10.02 ± 0.28	9.98 ± 1.21	1.24 ± 0.036	5.46 ± 0.36	1.46 ± 0.048

**Table 5:** Box–Behnken design of HC nanophytosome with variables and their levels obtained from Design Expert.

Independent Variable (Factor)	Factors Level		
	Low	Medium	High
X1 = Concentration of Leciva-S90 (mg)	50	225	400
X2 = Concentration Tween 80 (mg)	60	280	500
X3 = Hydration time (min)	30	45	60
X4 = Sonication time (min)	5	17.5	30
**Dependent variables (Response)**	**Desirability**		
Y1 = Particle size (nm) and PDI	Minimize		
Y2 = Zeta Potential (mV)	Maximize		
Y3 = Entrapment efficiency (%)	Maximize		
Y4 = Drug release (%)	Maximize		

**Table 6:** Point-prediction composition of the optimised NPs, the desirability of responses and their predicted values.

Optimised Formula Composition	Response, Confidence = 95%			
	Type	Desirability	Predicted Mean	Std Dev
X1 = Conc. Leciva-S90 (350 mg)	Y1 = particle size (nm) and PDI (%)	Minimize	105 nm and 26.9%	3.12
X2 = Conc. Tween 80 (460 mg)	Y2 = Zeta Potential (mV)	optimize	−15.13	4.53
X3 = Hydration time (30 min)	Y3 = Entrapment efficiency (%)	Maximize	73.89	1.17
X4 = Sonication time (10 min)	Y4 = Drug release (%)	Maximize	85.43	2.73

## Data Availability

Data are contained within the article.

## Supplementary Materials

The following are available online at https://www.mdpi.com/article/10.3390/ph17020213/s1, Supplementary file: Rheology of HC-NPsPO (gel formulations) and Particle size.

## List of Abbreviations

DSC	Differential Scanning Calorimetry
EE	Entrapment efficiency
ELISA	enzyme-linked immunoassay
HC	*Hedyotis corymbosa*
H&E	Hematoxylin and Eosin
IMQ	Imiquimod
IL	Interleukin
MCP-1	monocyte chemotactic protein 1
NPs	Nano-Phytosome
PBS	Phosphate buffer saline
PDI	Polydispersity index
TEM	Transmission electron microscopy
TNF	Tumour Necrosis Factor
