# Supplementary material for: Nanophytosomal Gel of Heydotis corymbosa (L.) Extract against Psoriasis: Characterisation, In Vitro and In Vivo Biological Activity
Source: Pharmaceuticals (Basel). 2024 Feb 6;17(2):213. doi: 10.3390/ph17020213 (PMC10893228; doi:10.3390/ph17020213)
Supplement: Supplementary file 1 [file pharmaceuticals-17-00213-s001.zip › Particle size.pdf]

Measurement information

**Measurement name**	Cholestrol	**User**	admin
**Method**	-	**Time**	1/21/2023 2:53:17 PM
**Status**	Succeeded	**Instrument type**	Litesizer 100
**Measurement mode**	Particle size		
		**Filter opical density**	3.461 (Automatic)
**Measurement cell**	Disposable	**Focus position**	-3.7 mm (Automatic)
**Measurement angle**	Back scatter (Manual)	**Material**	Unknown material
**Target temperature**	25.0 °C	**Material refractive index**	-
**Equilibration time**	0h 01m 00s	**Material absorbance coefficient**	-
**Analysis model**	General	**Solvent**	PBS
**Cumulant model**	Advanced	**Solvent refractive index**	1.3318
**Processed runs**	6 (Automatic)	**Solvent viscosity**	0.0009041 Pa.s
**Time for each run**	0h 00m 10s (Automatic)		

Particle size distribution (intensity)

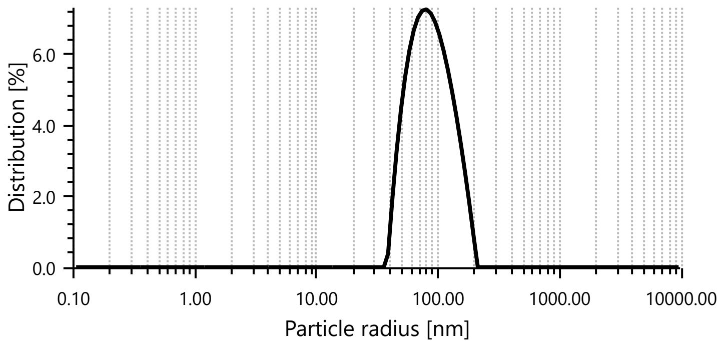

Results

**Hydrodynamic radius**	86.11 nm	**Mean intensity**	303.2 kcounts/s
**Polydispersity index**	22.6 %	**Absolute intensity**	876109.5 kcounts/s
**Diffusion coefficient**	2.8 µm²/s	**Intercept g1²**	0.8277
**Transmittance**	0.4 %	**Baseline**	1.001

Particle size distribution peaks (intensity)

Peak name	Size [nm]	Area [%]	Standard deviation [nm]
Peak 1	91.43	100.00	31.20
Peak 2	-	-	-
Peak 3	-	-	-
