# Supplementary material for: Nanophytosomal Gel of Heydotis corymbosa (L.) Extract against Psoriasis: Characterisation, In Vitro and In Vivo Biological Activity
Source: Pharmaceuticals (Basel). 2024 Feb 6;17(2):213. doi: 10.3390/ph17020213 (PMC10893228; doi:10.3390/ph17020213)
Supplement: Supplementary file 1 [file pharmaceuticals-17-00213-s001.zip › Rheology of gel.pdf]

Rheology of HC-NPsPO

HC-NPsPO exhibited a pseudoplastic flow behavior (Fig.1), which means that their viscosities decrease with an increase in shear rate. Pseudoplasticity or shear thinning of gel could be due to its colloidal network structure, which aligns itself in the direction of the shear and thereby decreases in the apparent viscosity when the shear rate increases. Moreover, pseudoplastic flow behavior displayed by HC-NPsPO could facilitate the spreadability of gels once shear stress is applied, which ensures the maximum area of coverage during gel application, whereas during static conditions, the gels are capable of returning to the viscous gel form. Therefore, the developed gel achieved the desirable and ideal requirement of the topical gels.

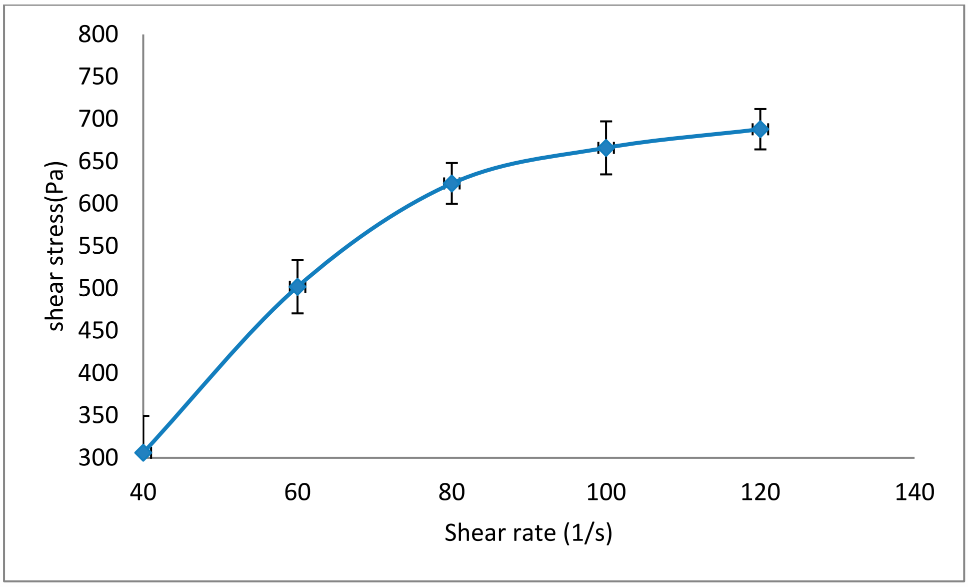

Figure S1: Rheological curve of flow HC-NPsPO

Viscosity measurements were carried out at 25 ± 0.1 ◦C temperature using a rheometer (Brookfield rheometer DV-II+ Pro, Mumbai, India). Briefly, the gel samples were allowed to rest for 5 min prior to analysis. Lower the viscometer into the gel until the surface of gel is level with the groove mark on the viscometer spindle (LV-3(63) was selected on trial-error basis). The shear stress vs shear rate curve with a shear rate range of 60-120 s−1 was obtained. It was used to determine viscosities. Viscosities are measured at different shear rate 60, 80, 100 and 120 sec-1 to determine the flow behavior of gel (Fig 2).

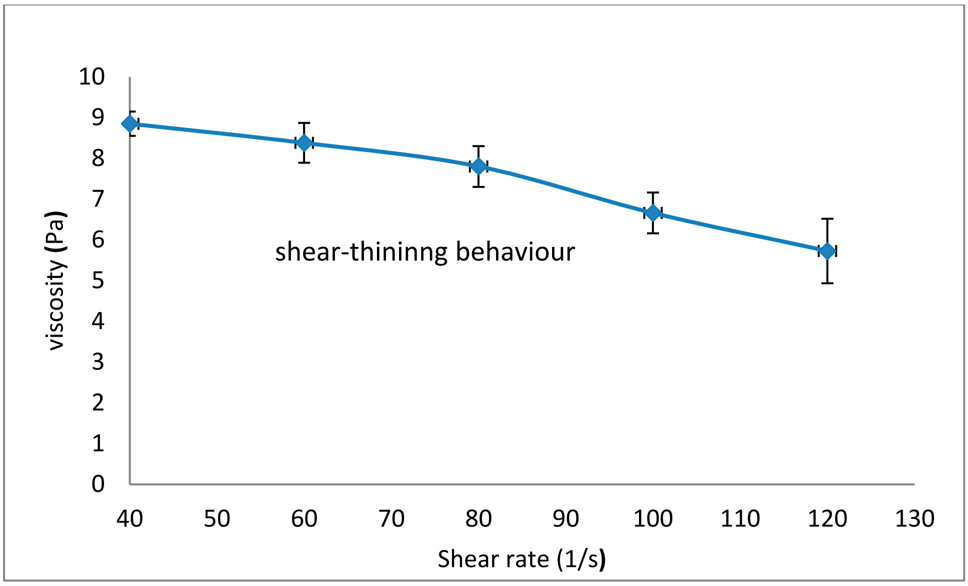

Figure S2: Rheological curve of viscosity HC-NPsPO
